# Impaired cerebrospinal fluid circulation and cerebral lymphatic drainage in a rat model of chronic hydrocephalus

**DOI:** 10.3389/fnmol.2025.1516265

**Published:** 2025-02-19

**Authors:** Dong Bin Back, Bo-Ryoung Choi, Kyoung Ja Kwon, Dong-Hee Choi, Chan Young Shin, Jongmin Lee, Hahn Young Kim

**Affiliations:** ^1^Department of Neurology, Research Institute of Medical Science, Konkuk University School of Medicine, Seoul, Republic of Korea; ^2^Department of Medicine, Research Institute of Medical Science, Konkuk University School of Medicine, Seoul, Republic of Korea; ^3^Department of Pharmacology, Research Institute of Medical Science, Konkuk University School of Medicine, Seoul, Republic of Korea; ^4^Department of Rehabilitation Medicine, Research Institute of Medical Science, Konkuk University School of Medicine, Seoul, Republic of Korea

**Keywords:** cerebrospinal fluid circulation, hydrocephalus, glymphatic, meningeal lymphatic, kaolin, animal model

## Abstract

The cerebrospinal fluid (CSF) not only protects the brain but also maintains homeostasis by removing metabolic waste produced by brain activity. This study hypothesizes that chronic CSF circulatory dysfunction, such as chronic hydrocephalus or normal pressure hydrocephalus (NPH), may be a critical condition in neurodegenerative diseases associated with metabolic waste accumulation. To investigate the CSF circulation and cerebral lymphatic drainage in a rat model of chronic hydrocephalus induced by kaolin injection, we performed time-dependent evaluations of intraparenchymal injection of tracers as well as intraventricular injection of Evans blue. The study systemically evaluated the dysfunction of CSF circulation and lymphatic drainage in the brain from various perspectives, including the glymphatic system, transependymal CSF flow, subarachnoid CSF flow, meningeal lymphatic drainage, and peripheral lymphatic drainage to deep cervical lymph nodes. The results indicated delayed CSF circulation, including glymphatic system, and cerebral lymphatic drainage in the kaolin-induced chronic hydrocephalus model. Based on these findings, our research indicated that dysfunction of CSF circulation, as observed in conditions such as chronic hydrocephalus or NPH, may act as an initiating or exacerbating factor in neurodegenerative diseases. This can lead to the accumulation of metabolic waste, as seen in Alzheimer’s disease. Our research can help identify risk factors and provide insight into the underlying pathophysiology of neurodegenerative diseases, which may lead to the development of novel therapeutic strategies.

## 1 Introduction

The human brain is surrounded by cerebrospinal fluid (CSF), which is primarily produced by the choroid plexus and circulates within the intraventricular and subarachnoid spaces, eventually being absorbed through the arachnoid granulations into the dural venous sinuses (Karimy et al., 2020). The CSF plays an important role in protecting the brain from physical trauma. However, the CSF plays an even more crucial role in interacting with the brain parenchyma, helping to maintain homeostasis by removing metabolic waste generated by brain activity (Karimy et al., 2020). Recent research has highlighted the perivascular space and meningeal lymphatic channels as pathways for metabolic waste clearance, providing a new perspective on the role of CSF circulation in the brain (Ahn et al., 2019, Rasmussen et al., 2018, Tarasoff-Conway et al., 2015).

In clinical practice, dysfunction of CSF circulation can manifest as communicating or non-communicating hydrocephalus depending on the presence of intraventricular obstruction (McAllister et al., 2015). Normal pressure hydrocephalus (NPH) is a type of communicating and chronic hydrocephalus characterized by increased CSF in the ventricles, often due to previous subarachnoid hemorrhage, infection, or head trauma, that chronically disrupts CSF circulation (Halperin et al., 2015, Karimy et al., 2020). However, it has been observed that many NPH patients do not have these pre-existing medical conditions, suggesting that NPH may be a manifestation of age-related or neurodegenerative dysfunction in CSF circulation (Silverberg et al., 2003). Impaired glymphatic clearance has been documented in NPH patients using MRI studies after intrathecal contrast agent injection (Rasmussen et al., 2018, Ringstad et al., 2017) or diffusion tensor imaging (Yokota et al., 2019). Furthermore, detailed identification of meningeal lymphatic drainage channels has been achieved in both rodent models (Ahn et al., 2019, Da Mesquita et al., 2018) and humans (Absinta et al., 2017, Zhou et al., 2020). The circulation of CSF seems to be tightly regulated by the complex interplay between intraparenchymal glymphatic clearance activity and meningeal lymphatic drainage.

In this study, our aim was to assess CSF circulatory dysfunction and cerebral lymphatic drainage from various perspectives, including the glymphatic system, transependymal CSF flow, subarachnoid CSF flow, meningeal lymphatic drainage, and peripheral lymphatic drainage to deep cervical lymph nodes. To achieve this, we used an animal model of chronic hydrocephalus induced by kaolin injection, a widely accepted method for replicating chronic hydrocephalus (Bloch et al., 2006, Klinge et al., 2003, Li et al., 2008, Silverberg et al., 2010, Silverberg et al., 2015) or NPH (Li et al., 2008) in rats. It was hypothesized that obstructing the intra-cisternal space through kaolin injection into the cisterna magna would impair CSF circulation.

To validate the relevance of the model to chronic hydrocephalus, we performed measurements of ventricular enlargement and behavioral tests such as beam walking test. Intraparenchymal glymphatic clearance was evaluated by analyzing the dispersion of intraparenchymally injected CSF tracers. In addition, unbound Evan blue (EB), injected intraventricularly, was tracked at various sites to assess transependymal flow, subarachnoid flow, meningeal lymphatic drainage, and peripheral lymphatic drainage to deep cervical lymph nodes, providing insight into CSF circulation pathways and cerebral lymphatic drainage.

Our investigation aimed to understand how clinical conditions such as chronic hydrocephalus or NPH, which impair CSF circulation, glymphatic clearance, and cerebral lymphatic drainage, may contribute to the progression of neurodegenerative diseases by impairing the mechanism responsible for removing metabolic waste.

## 2 Materials and methods

### 2.1 Animal preparation

Male Wistar rats (10 weeks-old, 300–320 g, Orient Bio) were used in all experiments after a 2 weeks acclimatization period in the Konkuk University vivarium before the start of the experiment. Rats were housed under standard laboratory conditions (22 ± 1°C temperature, 50 ± 10% humidity, 12 h alternate light/dark cycle) with *ad libitum* access to food and water. Animal experimental procedures were performed in accordance with the ethical approval of the Institutional Animal and Use Committee of Konkuk University and ARRIVE guidelines^1^. Rats were induced with 3% isoflurane and then anesthetized intraperitoneally with Zoletil 50 (30 mg/kg) and Rompun (10 mg/kg) before being secured in a stereotaxic frame. All stereotaxic injection experiments (kaolin with intra-cisterna magna injection, icm; CSF tracer with intraparenchymal injection; EB with intracerebroventricular injection, icv) had common preparation procedures, including anesthesia, head fixation in the stereotaxic frame, and reflow prevention by leaving the injection cannula in place for an additional 3 min, differing only in the specifications of the Hamilton syringe connected to the syringe pump (Harvard) and the needle size. Rats with kaolin-induced hydrocephalus were assigned to the CH group. Meanwhile rats with sham surgery were classified as the sham group.

### 2.2 Induction of kaolin-induced hydrocephalus

A midline incision was made to expose the atlanto-occipital membrane overlying the cisterna magna. A 60 μL of sterile suspension of kaolin (100 mg/mL in 0.9% saline, Sigma-Aldrich) was injected into the cisterna magna at a rate of 2.0 μL/s via a 28-gage cannula connected to polyethylene tubing, attached to a 100 μL Hamilton syringe with a 22-gage needle. Based on documented findings of normalized CSF dynamics (Brinker et al., 1998, Kondziella et al., 2002) and cerebral blood flow (Klinge et al., 2003), changes in cerebral metabolism (Klinge et al., 2003, Kondziella et al., 2002), cessation of ventricular enlargement (Braun et al., 1999, Klinge, 2011, Klinge et al., 2003), cognitive decline (Chen et al., 2017, Egawa et al., 2002) and behavioral changes (Hwang et al., 2009), our study established an 8 weeks post-kaolin infusion period for the development of a chronic hydrocephalus model in adult rats. All experimental evaluations were performed 8 weeks after kaolin induction of hydrocephalus.

### 2.3 Ventricular volume

Ventricular volumes were measured from multiple brain slices stained with cresyl violet acetate (Sigma-Aldrich) according to the methods described in our previous studies (Back et al., 2020, Back et al., 2017). Briefly, ventricular volumes, including the lateral and third ventricles, were calculated from the anterior horn of the lateral ventricle to the cerebral aqueduct using seven representative coronal slices at 2 mm intervals from bregma: AP +1.92 mm to −4.92 mm. Ventricular volume was calculated by multiplying the slice interval by the ventricular area obtained using the National Institutes of Health (NIH) Image J program.

### 2.4 Beam walking test

The beam walking test was performed as previously described (Allbutt and Henderson, 2007, Mu et al., 2011, Zhang et al., 2017), with minor modifications. Rats were assessed for their ability to traverse two parallel plastic beams, each 2.0 cm in diameter and 95 cm in length, positioned horizontally 50 cm above the ground. Prior to the kaolin icm (before 1 week), the rats participated in an adaptation training session to freely traverse the beams, eliminating any signs of fear or hesitation, and subsequent tests were conducted on consecutive days 1, 3, 7, 14, 28, 42, and 56 after the kaolin injection. After being placed on a platform (30 × 30 cm), we recorded the latency time and the number of footsteps (for each forelimb and hindlimb) for up to 60 s as the rats traversed from the moment their entire body entered the beam until they reached the opposite platform. The test began with 18 and 23 animals per group during the adaptation and early post-injection phases, but the sample size decreased over time, leaving three and four animals per group by the 8 weeks endpoint.

### 2.5 Intraparenchymal injection of cerebrospinal fluid tracers

We performed intraparenchymal CSF tracer injection experiments as described in our previous study (Back et al., 2020). Briefly, a 3 μL tracer solution containing Texas*-*red conjugated dextran (TR-d3, MW 3 kDa, Thermo Fisher Scientific) and fluorescein isothiocyanate (FITC) conjugated dextran (FITC-d40, MW 40 kDa, Sigma-Aldrich), both at 0.5% concentration in a 1:1 ratio, was dissolved in artificial CSF (aCSF, Tocris Bioscience). In the co-infusion experiment, the pre-prepared 3 μL tracer solution was administered into the brain parenchyma at a rate of 0.5 μL/min at bregma-related coordinates (AP +2.0 mm, ML +2.6 mm, DV −5.4 mm) using a 30-gage injection cannula connected to polyethylene tubing, attached to a 10 μL Hamilton syringe with a 26 s-gage needle. The infusion cannula was kept in place for 10 min to limit the backflow of tracer from the CSF. Six hours after injection, rats were transcardially perfused with 0.01 M phosphate-buffered saline, pH 7.4 (1x PBS) and fixed with 4% paraformaldehyde (PFA) for fluorescence imaging analysis.

### 2.6 Intracerebroventricular injection of EB

EB (DB 53, MW 0.96 kDA, Sigma-Aldrich) was suspended in 0.9% and injected into the ventricle saline (10 μL at 1.0 μL/min, 20 mg/mL) at coordinates relative to bregma (AP −0.9 mm, ML +1.4 mm, and DV −3.8 mm) using a 30-gage injection cannula connected to polyethylene tubing, attached to a 25 μL Hamilton syringe with a 22-gage needle. Similar to the CSF tracers, the cannula was left in place for approximately 10 min to prevent backflow. For imaging and quantitative analysis at 3 or 24 h after icv EB injection, rats were perfused with PBS, omitting the fixative solution, to facilitate neck incision for deep cervical lymph nodes harvesting, which was also used to obtain coronal brain slices and whole brain tissue to ensure experimental consistency. Meanwhile, EB-stained meninges were dissected without transcardiac perfusion of both PBS and PFA to preserve the integrity of meningeal lymphatic vessels.

### 2.7 Tissue preparation and immunohistochemistry

After isoflurane anesthesia and transcardiac perfusion, rat brains were immediately removed, post-fixed overnight in the same fixative, sequentially cryoprotected in 30% sucrose solution, and were embedded in an optimal cutting temperature (OCT) compound (Sakura Finetek). Brains were serially sectioned into 40 μm coronal slices using a CM1520 cryostat (Leica) for immunohistochemistry and tracer studies.

To obtain whole mounts of rat dural meninges, we adapted methods from previously reported mouse experiments (Bolte et al., 2023, Louveau et al., 2018, Louveau et al., 2015, Nilsson et al., 2022, Roussel-Queval et al., 2023), with minor modifications. Briefly, at 3 or 24 h after icv EB injection, anesthetized rats were decapitated above the shoulders, and the skin and muscle were removed from the outer skull. Using a bone cutter (Roboz), a precise dissection was made from the foramen magnum to the lateral end of the occipital bone, extending transversely in the longitudinal plane across the squamous and frontal bones to the junction of the frontal and nasal bones, taking care to minimize damage to the brain tissue. In order to obtain preserved meninges firmly attached to the adjacent skull, the skull bone was levered anteriorly outwards and meninges were gently separated from the bone using an elevator (Roboz). The separated EB-stained meninges, which were gently washed with PBS for 1 min to remove blood while retaining EB, were then used for immunohistochemistry and quantitative analysis of EB distribution.

To obtain deep cervical lymph nodes, rats perfused only with PBS underwent a midline incision (5 mm above the clavicle) to expose the sternocleidomastoid muscle, which was then retracted with forceps, followed by post-fixation, cryoprotection, and embedding procedures (similar to brain sections) for quantitative analyses of EB distribution.

After sample preparation, immunofluorescence staining was performed to assess various pathological outcomes. Brain sections (either intact or stained with EB) were washed (1x PBS/0.3% Triton X-100) and then incubated in blocking serum (1x PBS/10% normal donkey serum/0.3% Triton X-100) for 1 h at room temperature (RT), while the meninges started the incubation process. Double-label immunofluorescence was performed overnight at 4°C in a solution (1x PBS/0.15% normal donkey serum/0.3% Triton X-100) containing the following primary antibodies: mouse anti-glial fibrillary acidic protein (GFAP, 1:1000, BD Bioscience), rabbit anti-ionized calcium binding adapter molecule-1 (Iba-1, 1:1000, Wako), rabbit anti-collagen IV (COLIV, 1:100, Abcam), mouse anti-aquaporin 4 (AQP4, 1:100, Abcam), mouse anti-collagen IV (COLIV, 1:100, Sigma-Aldrich), rabbit anti-lymphatic vessel endothelial hyaluronan receptor 1 (LYVE1, 1:100, Abcam). The sections were then washed (1x PBS/0.15% Triton X-100) and incubated for 3 h at RT in a secondary antibody solution as follows: anti-mouse Alexa Fluor 488 and 568; anti-rabbit Alexa Fluor 488 and 568; all from donkey (1:200, Invitrogen). Some of the coronal sections were counterstained with TO-PRO-3 (1:1,000, Invitrogen) for 30 min at RT. Stained sections were mounted on slides, dried for 30 min, and coverslipped with ProLong^®^ Gold antifade reagent (Invitrogen), excluding structurally uneven meninges.

### 2.8 Aquaporin 4 polarization

Astrocytic AQP4 polarization was assessed according to our previous study (Back et al., 2017). Briefly, we selected six regions of interest—three in the left and three in the right corpus callosum, categorized as medial, middle, and lateral–each measuring 500 × 500 μm, spanning from the medial corpus callosum adjacent to the cingulate gyrus, through the middle, to the lateral corpus callosum for analysis. A low stringency threshold defined the total area of AQP4 immunoreactivity, whereas a high stringency threshold identified the vascular AQP4 colocalized with COLIV. Image J (NIH) was used for area calculations after black and white thresholding. The ratio of low stringency area to high stringency area was defined as AQP4 polarization.

### 2.9 Image analysis and quantification

The EB-stained area and intensity in the transependymal pathway were analyzed using a Carl Zeiss LSM 900 confocal laser scanning microscope with a 10 × objective (air, NA 0.45) equipped with the Zeiss tile function to capture consecutive tiles covering entire brain regions. Bright-field photographs of the four different (caudal, ventral, lateral, dorsal) brain surfaces, focusing on the major pathway stained with EB, were captured using a digital camera. A standard exposure time was applied consistently across all samples to ensure uniform imaging conditions. Immunofluorescence images were obtained using the same Zeiss microscope at different magnifications (10 × air, NA 0.45; 20 × air, NA 0.80; 40 × water, NA 1.2; 63 × oil, NA 1.4) with a resolution of 1,024 × 1,024 pixels, acquired by multichannel scanning in either a single or tiled frame. Capillary filling of EB into the microvessels in the corpus callosum was analyzed both by z-stack three-dimensional reconstruction (image slices taken at 22 μm intervals consisting of 12 z-sections), with upper and lower limits set based on positively visualized vascular morphology for COLIV and AQP4, and by fluorescence co-localization in the XY and orthogonal XZ, YZ projections of the image stacks. Image analysis and quantification was performed using Image J (NIH) and Zen blue image analysis wizard (Carl Zeiss).

### 2.10 Statistics

Behavioral data were analyzed using two-way repeated measures analysis of variance (ANOVA) and unpaired *t*-tests. Two-way ANOVA was used to assess the interaction between group and time, followed by a post-hoc Tukey’s honest comparison to analyze differences between groups over time. Time or group dependent differences were analyzed using unpaired *t*-tests, where applicable. Pearson’s correlation coefficient (r) was used for correlation analyses between ventricular volume and other variables. All data are expressed as the mean ± SEM. A value of *p* < 0.05 was considered to be statistically significant. Data analysis was performed with SPSS software version 27.0.

## 3 Results

### 3.1 Characteristics of rats with chronic hydrocephalus

**FIGURE 1:**
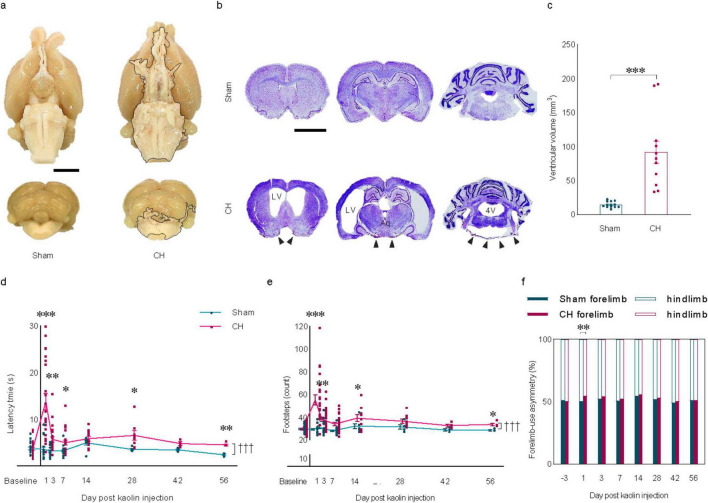
Induction of chronic hydrocephalus via intra-cisterna magna kaolin injection and resulting behavioral changes. **(A)** Representative brain regions showing kaolin deposition indicated by the black lining in the CH group. **(B)** Representative cresyl violet stain depicting ventricular enlargement with kaolin-contacted inflammation indicated by black arrowheads in the CH group. **(C)** Quantification of ventricular volume, *n* = 7–10. **(D)** Latency time and **(E)** footsteps, and **(F)** forelimb-use asymmetry in the beam walking test, *n* = 18–23 up to 1 week; *n* = 7–11 at 2 weeks; *n* = 5–8 at 4 weeks; *n* = 3–4 up to 8 weeks. Scale bar = 2 mm; *p < 0.05, **p < 0.01, ***p < 0.001; ^†††^*p* < 0.001; CH, chronic hydrocephalus; LV, lateral ventricle; Aq, cerebral aqueduct; 4V, fourth ventricle.

Eight weeks after icm injection of kaolin, the extracted brains showed a nearly uniform distribution of kaolin deposits throughout the ventral regions, extending from the optic nerves to the medulla and covering the interpeduncular fossa, pons, and the posterior cerebellum as indicated by the black lining (Figure 1A). Due to its higher density compared to CSF, it is minimally affected by CSF flow and did not get swept along with the circulating CSF. Kaolin-contacted inflammation as indicated by black arrowheads in the CH group (Figure 1B). Marked hydrocephalus was observed, characterized by symmetrical enlargement of the lateral ventricles, cerebral aqueduct, and the fourth ventricle (Figure 1B) with a quantitatively significant increase in total ventricular volume in the CH group (*p* < 0.001, Figure 1C).

Modified beam walking test showed a significant difference in patterns (F_(7,273)_ = 15.359, *p* < 0.001, latency time in Figure 1D; F_(7,273)_ = 5.664, *p* < 0.001, the number of footsteps in Figure 1E). At the various time points, there was a significant increase in latency time (*p* < 0.001, post-op day 1; *p* < 0.01, post-op day 3, 56; *p* < 0.05, post-op day 7, 28; Figure 1D) and the number of footsteps (*p* < 0.001, post-op day 1; *p* < 0.01, post-op day 3; *p* < 0.05, post-op day 14, 56; Figure 1E) in the CH group compared to the sham group. A more frequent use of the forelimbs than the hindlimbs was observed in the CH group compared to the sham group on post-op day 1 (*p* < 0.01, Figure 1F). The gait impairment in the CH group was pronounced within the first 7 days post-operation, with the impairment persisting for the entire 8 weeks period, suggesting a chronic motor function deficit in rats with kaolin-induced hydrocephalus.

### 3.2 Neuroinflammation associated with kaolin-induced chronic hydrocephalus

**FIGURE 2:**
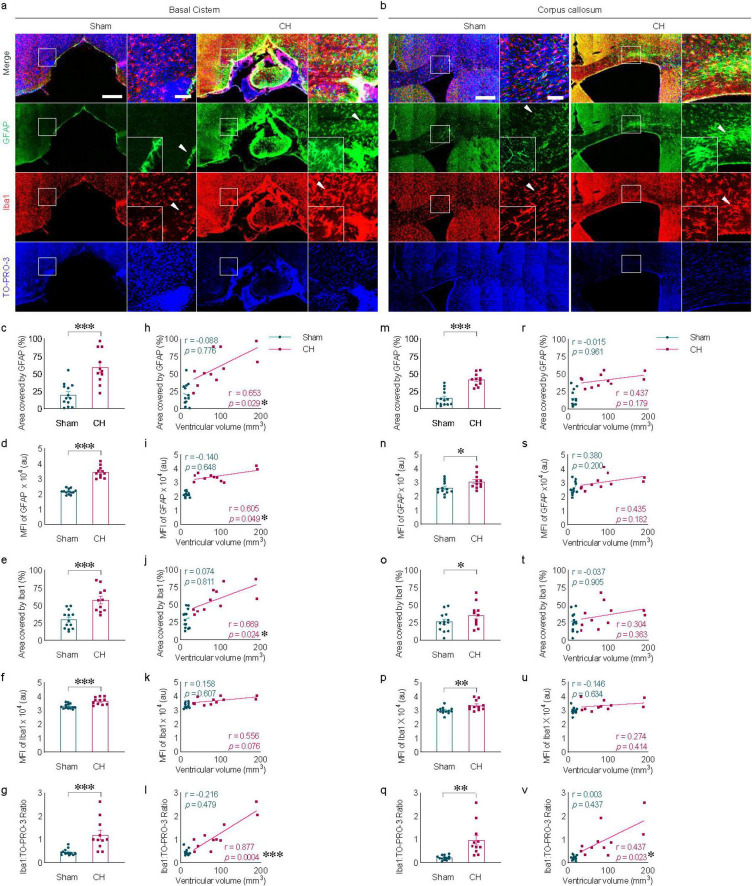
Neuroinflammation in kaolin-induced chronic hydrocephalus. Neuroinflammation in the basal cistern **(A)** or corpus callosum **(B)** measured by GFAP and Iba1, quantified by covered area [**(C,M)** in GFAP, **(E,O)** in Iba1], by fluorescence intensity [**(D,N)** in GFAP, **(F,P)** in Iba1], or Iba1/TO-PRO-3 ratio **(G,Q)** and its correlation with ventricular volume [**(H–L)** and **(R–V)**]. *n* = 11–13; scale bar = 500 μm (100 μm in the inset); *p < 0.05, **p < 0.01, ***p < 0.001; CH, chronic hydrocephalus; MFI, mean fluorescence intensity; au, arbitrary unit.

Meningeal fibrosis, characterized by chemical inflammatory arachnoiditis in the basal cistern due to direct contact with kaolin (Figure 2A) and indirect inflammation in the corpus callosum influenced by ventricular enlargement (Figure 2B) are notable features in the CH group. Quantitative analysis revealed a significant increase in reactive astro- and microgliosis within the CH group, measured by area (Figures 2C, M in GFAP; Figures 2E, O in Iba1), fluorescence intensity (Figures 2D, N in GFAP; Figures 2F, P in Iba1), and the ratio of the number of Iba1-positive cells to total number of TO-PRO-3-positive cells (Figures 2G, Q). Furthermore, within the CH group, a positive correlation was observed between ventricular volume and GFAP signal measured by area (r = 0.653, *p* < 0.05, Figure 2H) and fluorescence intensity (r = 0.605, *p* < 0.05, Figure 2I), as well as Iba1 signal measured by area in the basal cistern (r = 0.669, *p* < 0.05, Figure 2J). In addition, the Iba1 to TO-PRO-3- positive cell ratio in the basal cistern (r = 0.877, *p* < 0.001, Figure 2L) and corpus callosum (r = 0.437, *p* < 0.05, Figure 2V) correlated with ventricular volume, suggesting a positive relationship between ventricular dilatation and the severity of neuroinflammation. However, in certain instances, no statistically significant correlation was found between ventricular volume and neuroinflammation markers (Figures 2K, R–U).

### 3.3 AQP4 depolarization in the white matter in rats with chronic hydrocephalus

**FIGURE 3:**
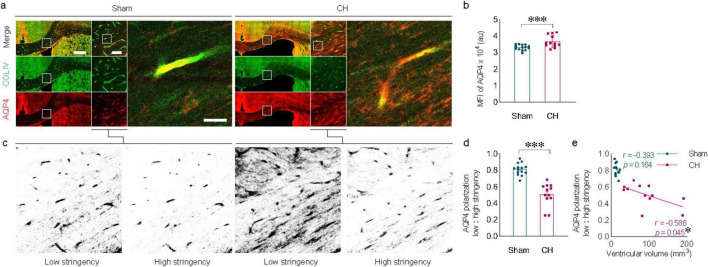
Alterations in AQP4 expression and AQP4 depolarization. **(A)** Representative images of AQP4 expression in the corpus callosum, depicting depolarization of AQP4 in the CH group. **(B)** Quantification of AQP4 expression. **(C)** Schematic diagram illustrating the calculation of AQP4 polarization using low and high stringency. **(D)** AQP4 polarization index. **(E)** Correlation between AQP4 polarization index and ventricular volume. *n* = 12–14; scale bar = 500 μm (100 μm in the inset); *p < 0.05, ***p < 0.001; CH, chronic hydrocephalus; MFI, mean fluorescence intensity; au, arbitrary unit.

Changes in AQP4 expression were evident in the corpus callosum of the CH group (Figure 3A). In addition to the increase in parenchymal AQP4 expression (*p* < 0.001, Figure 3B), the distribution pattern of AQP4 also changed, indicated by redistribution into the brain parenchyma from the location on perivascular astrocyte endfeet in close contact with COLIV-positive endothelial vessels (Figure 3C). Perivascular AQP4 localization, indicative of the AQP4 polarization ratio, was significantly reduced in the CH group compared to the sham group (*p* < 0.001, Figure 3D). This loss of AQP4 polarization also correlated well with ventricular dilatation (r = −0.586, *p* < 0.05, Figure 3E).

### 3.4 Delayed dispersion of intraparenchymally injected tracers in rats with chronic hydrocephalus

**FIGURE 4:**
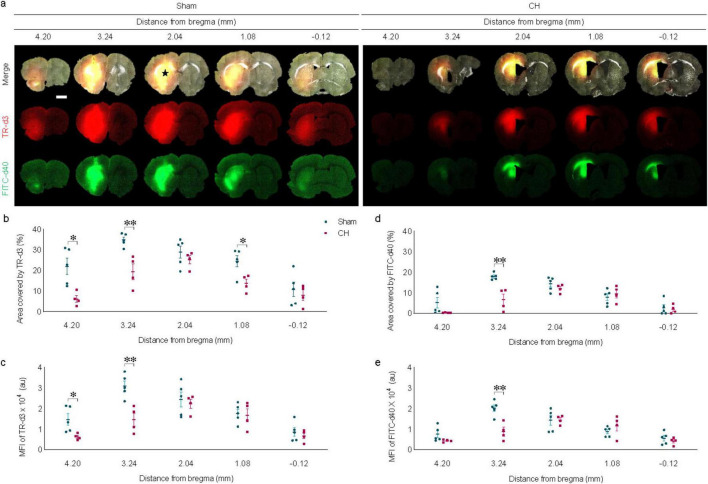
Dispersion of intraparenchymally injected tracers. **(A)** Representative images illustrating intraparenchymal dispersion of CSF tracers at 6 h post-injection, quantified by covered area **(B,D)** or by fluorescence intensity **(C,E)**. The black star in **(A)** indicates the intraparenchymal tracer injection site. *n* = 4–5; scale bar = 2 mm; *p < 0.05, **p < 0.01; CH, chronic hydrocephalus; TR-d3, Texas-red conjugated dextran; FITC-d49, fluorescein isothiocyanate conjugated dextran; MFI, mean fluorescence intensity; au, arbitrary unit.

Intraparenchymally injected tracers of different molecular weights exhibited delayed dispersion patterns at 6 h post-injection in the CH group compared to the sham group (Figure 4A). The black star in Figure 4A indicates the intraparenchymal tracer injection site. Tracer dispersion into the brain parenchyma was impaired in the CH group in multiple brain slices in both cases of TR-d3 (*p* < 0.05 at 4.20 mm and 1.08 mm, *p* < 0.01 at 3.24 mm, assessed by area in Figure 4B; *p* < 0.05 at 4.20 mm, *p* < 0.01 at 3.24 mm, assessed by fluorescence intensity in Figure 4C) and FITC-d40 (*p* < 0.05 at 3.24 mm, assessed by area in Figure 4D; *p* < 0.01 at 3.24 mm, assessed by fluorescence intensity in Figure 4E). The lighter TR-d3 tracer exhibited greater dispersion both anteriorly and posteriorly from the injection site than the heavier FITC-d40 tracer.

### 3.5 Transependymal and subarachnoid dispersion of icv-injected EB in rats with chronic hydrocephalus

**FIGURE 5:**
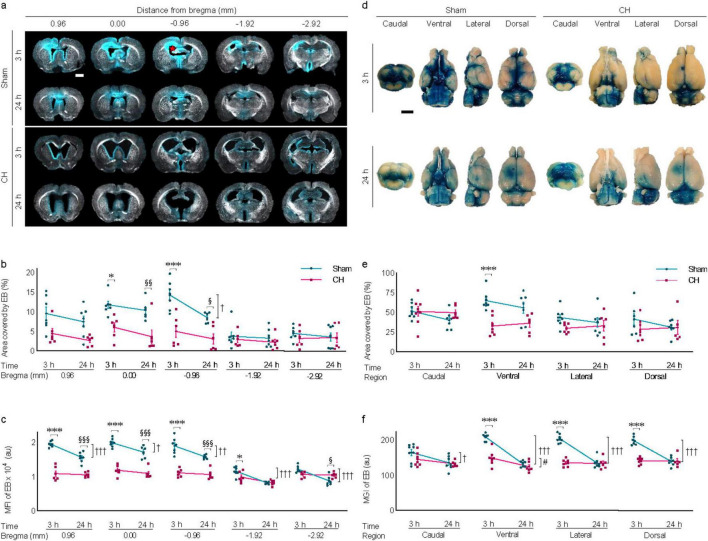
Transependymal and subarachnoid dispersion of icv injected EB. **(A)** Representative images illustrating time-dependent transependymal dispersion of EB, quantified by covered area **(B)** or by fluorescence intensity **(C)**, The red star in **(A)** indicates the icv EB injection site. *n* = 6–7. **(D)** Representative images illustrating time-dependent subarachnoid dispersion of EB, quantified by covered area **(E)** or by fluorescence intensity **(F)**, *n* = 6–8. Scale bar = 2 mm; *p < 0.05, ***p < 0.001 (CH 3 h vs. Sham 3 h); ^†^*p* < 0.05, ^††^*p* < 0.01, ^†††^*p* < 0.001 (Sham 3 h vs. Sham 24 h); #*p* < 0.05 (CH 3 h vs. CH 24 h); ^§^*p* < 0.05, ^§§^*p* < 0.01, ^§§§^*p* < 0.001 (Sham 24 h vs. CH 24 h); icv, intracerebroventricular injection; EB, Evans blue; CH, chronic hydrocephalus; MFI, mean fluorescence intensity; MGI, mean gray intensity; au, arbitrary unit.

Transependymal dispersion of icv-injected EB was visibly present at 3 hours post-injection and disappeared by 24 h in the sham group, whereas it was scarce in the CH group (Figure 5A). The red star in Figure 5A indicates the icv EB injection site. Five sections around the injection site were selected to assess the pattern of dispersion depending on time or model. In multiple sections, transependymal dispersion was significantly impeded in the CH group compared to the sham group throughout the 24 h when assessed by the EB-stained area (*p* < 0.05 at 0.00 mm, *p* < 0.001 at −0.96 mm, CH 3 h vs. Sham 3 h; *p* < 0.01 at 0.00 mm, *p* < 0.05 at −0.96 mm, CH 24 h vs. Sham 24 h; Figure 5B) and fluorescence intensity (*p* < 0.001 at 0.96, 0.00, and −0.96 mm, *p* < 0.05 at −1.92 mm, CH 3 h vs. Sham 3 h; *p* < 0.001 at 0.96, 0.00, and −0.96 mm, *p* < 0.05 at −2.92 mm, CH 24 h vs. Sham 24 h; Figure 5C). While the sham group showed a time-dependent washout pattern with a 3 h-prominence post-injection and a subsequent decrease at 24 h (*p* < 0.05 at −0.96 mm, Sham 3 h vs. Sham 24 h, assessed by area in Figure 5B; *p* < 0.05 at 0.00 mm, *p* < 0.01 at −0.96 mm, *p* < 0.001 at 0.96, −1.92 and −2.92 mm, Sham 3 h vs. Sham 24 h, assessed by fluorescence intensity in Figure 5C), the CH group showed minimal initial dispersion into the transependymal region and a persistent pattern.

Subarachnoid dispersion of icv-injected EB was also hindered in the CH group throughout the 24 h compared to the sham group (Figure 5D). EB staining was quantified by assessing views from the four different angles including caudal, ventral, lateral, and dorsal angels. Subarachnoid dispersion was significantly impeded in the CH group compared to the sham group when assessed by the EB-stained area (*p* < 0.001 at ventral angle, CH 3 h vs. Sham 3 h in Figure 5E) and fluorescence intensity (*p* < 0.001 at ventral, lateral, and caudal angles, CH 3 h vs. Sham 3 h in Figure 5F). The time-dependent washout pattern was observed in the sham group (*p* < 0.05 at caudal, *p* < 0.001 at ventral, lateral, and dorsal angles, Sham 3 h vs. Sham 24 h, assessed by fluorescence intensity in Figure 5F), whereas it was not evident in the CH group except at ventral angle (*p* < 0.05, assessed by fluorescence intensity in Figure 5F).

### 3.6 Transependymal capillary filling of icv-injected EB in rats with chronic hydrocephalus

**FIGURE 6:**
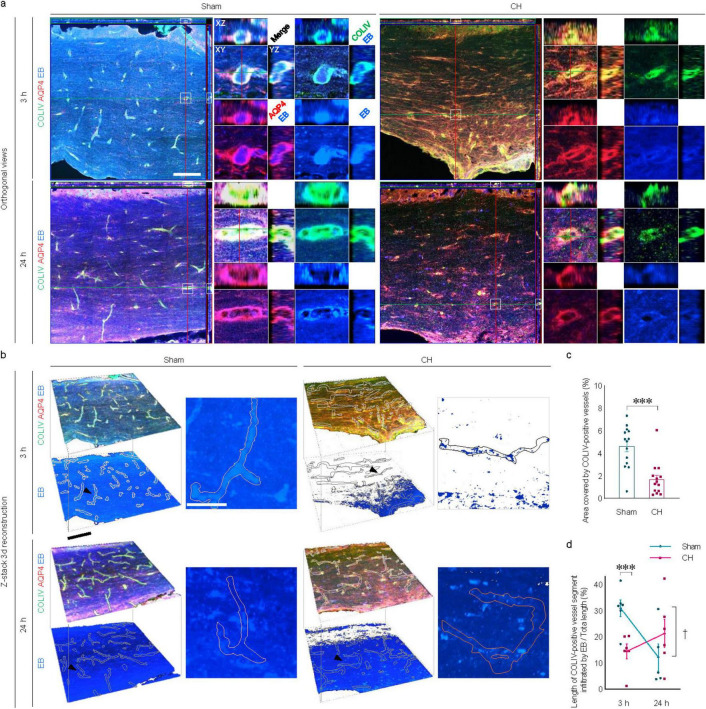
Transependymal capillary filling of icv injected EB. Representative images illustrating time-dependent capillary filling of EB in the corpus callosum imaged by confocal immunofluorescence with orthogonal view **(A)** and three-dimensional reconstruction from z-stacks **(B)**. Single capillary vessels focused with a black arrowhead in **(B)** are magnified as insets. **(C)** Quantification of capillary densities, ***p < 0.001. **(D)** Time-dependent profiles of capillary filling, ***p < 0.001 (CH 3 h vs. Sham 3 h); ^†^*p* < 0.05 (Sham 3 h vs. Sham 24 h). *n* = 14; Scale bar = 100 μm (50 μm in the inset); CH, chronic hydrocephalus; icv, intracerebroventricular injection; EB, Evans blue.

Examination by confocal microscopy revealed apparent transependymal capillary filling of the icv-injected EB in the corpus callosum of the sham group, whereas it was impaired in the CH group (orthogonal views in Figure 6A and z-stack 3D in Figure 6B). Capillary density measured by COLIV-positive blood vessels within the unit region (%) showed a significant decrease in the CH group compared to the sham group (*p* < 0.001, Figure 6C).

In the sham group, the capillary filling of the EB, which was evident at 3 h post-injection, disappeared at 24 h. In contrast, transependymal capillary filling of EB in the CH group was minimal throughout the 24 h. Quantitative evaluation using the ratio of EB-filled capillary length to the total capillary length showed peaks at 3 h, indicating rapid capillary filling in the sham group compared to the CH group (*p* < 0.001, CH 3 h vs. Sham 3 h, Figure 6D), and declines at 24 h, indicating subsequent capillary emptying (*p* < 0.05, Sham 3 h vs. Sham 24 h, Figure 6D). In contrast, the CH group showed peaks at 24 h instead of at 3 h, indicating delayed capillary filling.

### 3.7 Meningeal lymphatic drainage in rats with chronic hydrocephalus

**FIGURE 7:**
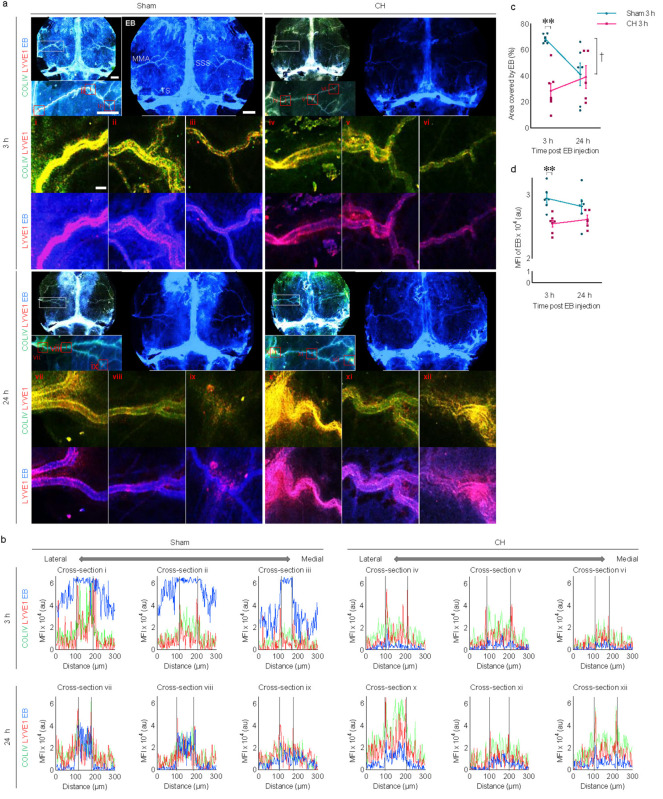
Meningeal lymphatic drainage of icv injected EB. **(A)** Representative images illustrating time-dependent EB staining of whole-mount meninges, co-stained with COLIV (meningeal vasculature) and LYVE1 (meningeal lymphatics). White boxes are focused on the middle meningeal arteries and further magnified with red boxes (i–xii). **(B)** Cross-section analysis of the meningeal lymphatic vessels with multi-signal intensities of COLIV/LYVE1/EB in the red boxes (i–xii). The lumen of meningeal lymphatic vessels is indicated by black dotted lines in **(B)**. Meningeal EB staining quantified by covered area **(C)** or mean fluorescence intensity **(D)**. **p < 0.01 (CH 3 h vs. Sham 3 h); ^†^*p* < 0.05 (Sham 3 h vs. Sham 24 h). *n* = 11–13; scale bar = 2 mm (100 μm in the red boxes); CH, chronic hydrocephalus; icv, intracerebroventricular injection; EB, Evans blue; SSS, superior sagittal sinus; TS, transverse sinus; MMA, middle meningeal artery; MFI, mean fluorescence intensity; au, arbitrary unit.

Immunohistochemistry using whole-mount meninges revealed the colocalization of scattered lymphatic endothelial signals (LYVE1) alongside meningeal vascular signals (COLIV), as shown in Figure 7A. Cross-sectional analysis of multi-immunofluorescence signals showed red peaks (LYVE1) surrounding green peaks (COLIV) in Figure 7B. After 3 h of icv-injected EB in the sham group, distinct blue peaks (EB) were observed between the two red peaks, as shown by the black dotted lines indicating the lumen of meningeal lymphatic vessels in Figure 7B, suggesting effective EB influx into the meningeal lymphatic vessels. This distinct pattern of blue peaks disappears at post-24 h of icv-injected EB in the sham group, indicating subsequent efficient drainage of EB via meningeal lymphatic vessels. In contrast to the sham group, the CH group showed no clear influx of EB into the meningeal lymphatic vessels and subsequent drainage.

Quantitative analysis using EB signals on the meninges revealed a consistent impairment of meningeal lymphatic drainage in the CH group throughout the 24 h compared to the sham group. This impairment is evident in the CH group when assessing CH 3 h vs. Sham 3 h, as indicated by the EB-stained area (*p* < 0.01, Figure 7C) or fluorescence intensity (*p* < 0.01, Figure 7D). In contrast to the normal meningeal lymphatic drainage pattern in the sham group, which peaked at 3 h and then disappeared at 24 h (*p* < 0.05, Sham 3 h vs. Sham 24 h, Figure 7C), the CH group showed minimal influx into the meningeal lymphatic vessels and a stagnant pattern.

### 3.8 Peripheral lymphatic drainage in rats with chronic hydrocephalus

**FIGURE 8:**
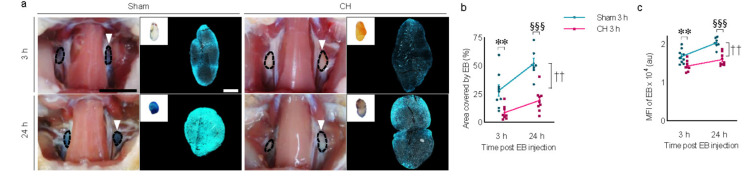
Peripheral lymphatic drainage of icv injected EB into the deep cervical lymph nodes. **(A)** Representative images illustrating time-dependent EB-stained deep cervical lymph nodes (white arrowheads) and magnified with fluorescence images, quantified by covered area **(B)** or mean fluorescence intensity **(C)**. *n* = 7–9; scale bar = 1 cm (100 μm in the inset); **p < 0.01 (CH 3 h vs. Sham 3 h); ^††^*p* < 0.01 (Sham 3 h vs. Sham 24 h); ^§§§^*p* < 0.001 (Sham 24 h vs. CH 24 h); CH, chronic hydrocephalus; icv, intracerebroventricular injection; EB, Evans blue; MFI, mean fluorescence intensity; au, arbitrary unit.

Time-dependent peripheral lymphatic drainage of icv-injected EB into the deep cervical lymph nodes is shown in both groups. Comparing the two groups at different time points, EB signals were evident at 3 h post-injection and peaked at 24 h in the sham group, while a relatively weaker signal was observed in the CH group (Figure 8A). Peripheral lymphatic drainage of EB was consistently impaired in the CH group compared to the sham group throughout the 24 h (*p* < 0.01, CH 3 h vs. Sham 3 h; *p* < 0.001, CH 24 h vs. Sham 24 h, assessed by area in Figure 8B; *p* < 0.01, CH 3 h vs. Sham 3 h; *p* < 0.001, CH 24 h vs. Sham 24 h, assessed by fluorescence intensity in Figure 8C). Although both groups showed a time-dependent increase in peripheral lymphatic drainage of EB to the deep cervical lymph nodes, this was more pronounced in the sham group (*p* < 0.01, Sham 3 h vs. Sham 24 h, assessed by area in Figure 8B or fluorescence intensity in Figure 8C).

## 4 Discussion

The experimental findings using the kaolin-induced hydrocephalus rat model demonstrate how chronic hydrocephalus can disrupt the dynamics of CSF circulation associated with the glymphatic system and cerebral lymphatic drainage. It is hypothesized that the injection of kaolin into the rat cisterna magna, which limits CSF absorption through the subarachnoid space, could serve as a reliable model for studying clinical conditions such as chronic hydrocephalus or NPH, a communicating type of hydrocephalus. Although we acknowledge that the kaolin-induced hydrocephalus model, in which the CSF absorption pathway is artificially obstructed by injected kaolin, may not perfectly mimic the pathophysiology and clinical features of chronic hydrocephalus or NPH, we have modified our experimental settings to resemble chronic hydrocephalus or NPH observed in humans. The aim was to create a chronic and mild condition similar to NPH without directly obstructing the intraventricular circulation of CSF. To achieve this, we used a minimal amount of kaolin and prolonged the duration of the hydrocephalus.

Injection of kaolin into the cisterna magna is known to induce inflammation, which leads to meningeal fibrosis, specifically chemical arachnoiditis (Kondziella et al., 2002, Li et al., 2008, Nagra et al., 2008, Nagra et al., 2010). The majority of the kaolin deposits were distributed throughout the basal subarachnoid space, evenly on both sides of the ventral brainstem, including the interpeduncular fossa to the medulla and pons. No significant presence of kaolin was observed within the intraventricular system, particularly blocking the outlet of the fourth ventricle. The icv-injected EB diffused throughout the entire subarachnoid space through the outlet of the fourth ventricle. This diffusion pattern of EB indicates that CSF flow through the intraventricular and subarachnoid spaces was not mechanically obstructed in the kaolin-induced chronic hydrocephalus rat model.

When discussing hydrocephalus, it is important to differentiate between acute and chronic conditions based on duration. The kaolin-induced hydrocephalus model is a well-known animal model for chronic hydrocephalus as it develops and progresses hydrocephalus over several weeks to months (Brinker et al., 1998, Klinge, 2011, Klinge et al., 2003, Kondziella et al., 2002, Silverberg et al., 2015). Injected kaolin in the basal subarachnoid space can cause acute hydrocephalic symptoms within 2 weeks, leading to an increase in resistance to CSF outflow and subsequent elevation of intracranial pressure (Brinker et al., 1998, Kondziella et al., 2002). As hydrocephalus progresses from the acute phase to the chronic phase over a longer period of four to 6 weeks or more, the resistance to CSF outflow and intracranial pressure undergo changes (Brinker et al., 1998, Kondziella et al., 2002). Animal studies using MRI have shown ventricular enlargement, with a peak at 6 weeks, followed by a subsequent decline at 10 weeks (Klinge, 2011). The progressive increase in ventricular size up to 8 weeks reaches a new steady state characterized by the restoration of normal intracranial pressure and an increase in resistance to CSF outflow (Braun et al., 1999). Moreover, the normalization of cerebral blood flow after 8 weeks indicates that the post-operative 8 weeks time point we chose for evaluation is optimal for investigating chronic hydrocephalus, such as NPH (Klinge et al., 2003). Our model also identified gait impairments, which are clinical manifestations observed in human NPH (Halperin et al., 2015). The beam walking test was performed with a statistically adequate sample size during the initial and acute phases of hydrocephalus. However, practical constraints, including variability in disease progression and ethical concerns to minimize animal stress during the chronic phase, resulted in a reduction in sample size. Consequently, while statistical significance was observed between the two groups in the chronic phase, further investigation is warranted. Although the kaolin-induced hydrocephalus rat model has inherent limitations, it has potential for investigating the impact of chronic hydrocephalus on the glymphatic system and cerebral lymphatic drainage in neurodegenerative diseases, particularly in relation to the accumulation of metabolic waste.

The kaolin-induced hydrocephalus rat model’s pathophysiology is mainly focused on neuroinflammatory changes and alterations of AQP4, the brain’s most abundant water channel that regulates water homeostasis (Hasan-Olive et al., 2018). Neuroinflammation with gliosis in the basal forebrain and corpus callosum may be indirectly triggered by increased pressure in the subarachnoid or intraventricular space, in addition to the reactive gliosis due to direct contact with kaolin. In particular, TO-PRO-3 staining labels cells with compromised membranes, such as those undergoing necrosis or apoptosis, including neurons and glial cells in the hydrocephalus model. By comparing with Iba1 staining for microglia, we can assess the extent of cell damage and the effects of hydrocephalus progression. The degree of gliosis is strongly correlated with enlarged ventricular volume, which may indirectly support the possibility of pressure-related gliosis. A significant depolarization of AQP4 was observed, resulting in the translocation of the normal perivascular pattern of AQP4 to the parenchymal pattern with an increased total AQP4 signal in the CH group. The evidence indicates that neuroinflammation and depolarization of AQP4 play a significant role in the pathophysiology of impaired CSF circulation in chronic hydrocephalus.

Previous studies have reported impaired glymphatic system associated with the depolarization of AQP4 (Back et al., 2020, Back et al., 2017). In this study, we investigated the glymphatic efflux of intraparenchymally injected tracers with two different molecular weights. As anticipated, the molecular weight-dependent intraparenchymal dispersion of the tracers at 6 hours post-injection was hindered in the CH group, indicating a compromised glymphatic system in the chronic hydrocephalus.

Previous studies have evaluated the classical CSF circulation pathway, which involves production in the choroid plexus, intraventricular circulation, and absorption into the dural venous sinuses through the arachnoid villi (Bradley, 2015, Silverberg et al., 2003). To assess this pathway, we measured the subarachnoid dispersion of icv-injected EB. Analysis of the subarachnoid brain surface area covered by EB in the sham group revealed rapid subarachnoid dispersion at 3 h post-injection, which was subsequently eliminated by 24 h post-injection. In contrast, the CH group exhibited delayed and stagnated dispersion into the subarachnoid space, indicating a disturbance of the classical CSF circulation pathway in the chronic hydrocephalus.

Furthermore, we investigated an alternative transependymal pathway for CSF circulation. This pathway was discovered in a study that utilized precise MRI mapping of CSF flow with a tracer injected into the ventricle of healthy rats (Magdoom et al., 2019). The investigation identified previously unrecognized parenchymal perivascular space connections that spread across various brain regions, facilitating the direct transport of CSF from the ventricles to the subarachnoid space (Magdoom et al., 2019). In studies of hydrocephalus in humans, the absorption of CSF by periventricular tissues serves as a compensatory mechanism for increased intracranial pressure and sheds light on an alternative pathway for CSF circulation (Edwards et al., 2004, Gibbs and Tanenbaum, 2018, Wang et al., 2020). Consistent with these studies, our analysis of coronal brain slices stained by EB revealed rapid periventricular diffusion of icv-injected EB at 3 h post-injection and subsequent elimination at 24 h post-injection in the sham group, indicating active transependymal CSF flow in the normal condition. Conversely, minimal periventricular diffusion of EB was observed in the CH group. However, despite differences in the diffusion patterns of the CSF tracers and EB over time between the sham and CH models, we did not perform additional validation to confirm whether equal amounts of these fluorescent tracers were successfully delivered after injection. Despite the pathological condition in the CH model, the distribution of the tracers was expected to be comparable between both groups at earlier time points. Therefore, it was important to verify through the initial diffusion patterns to ensure that the amount of tracers injected into the setting actually entered the brain. This is recognized as a limitation of our study.

Based on the confocal microscopic image of the corpus callosum in the sham group, the capillaries were completely filled with EB three hours after injection and showed clear drainage within 24 h after injection. The microvascular density was found to be deceased in the CH group compared to the sham group, which is consistent with previous studies reporting a decrease in microvascular density in chronic hydrocephalus (Del Bigio and Bruni, 1988, Jones et al., 1991, Ulfig et al., 2004). In the CH group, there was a decrease in capillary density and no visible diffusion of EB into the capillaries even after 24 h. These findings suggest a disturbance of the alternative transependymal CSF circulation pathway in chronic hydrocephalus, as evidenced by compromised periventricular diffusion and capillary drainage of EB.

In the animal study, the final step of CSF efflux into the venous sinuses through the arachnoid villi was demonstrated using serial high resolution MRI tracking of injected tracer into the lateral ventricles (Magdoom et al., 2019). Time-dependent EB drainage through the venous sinus was observed in the sham group, supporting the classical CSF circulation pathway via venous sinus drainage. Analysis of EB staining on the meninges showed strong staining around the venous sinuses and the middle meningeal artery.

Recent research has also revealed the presence of lymphatic vessels in the meninges responsible for draining CSF, interstitial fluid, macromolecules, and immune cells to the cervical lymph nodes (Louveau et al., 2015, Yankova et al., 2021). In a previous study using novel lymphatic reporter rats, it was demonstrated that meningeal lymphatic vessels were located alongside the middle meningeal artery, superior sagittal sinus, and transverse sinuses (Jung et al., 2017). In our study, we used a whole-mount dissection of the dura mater and found that LYVE1-positive meningeal lymphatic vessels appeared as discontinuous dotted lines scattered along the COLIV-positive meningeal arteries. Cross-sectional analysis of multi-immunofluorescence signals revealed intraluminal filling and subsequent drainage of EB through the meningeal lymphatic vessels as a CSF efflux pathway. In the CH group compared to the sham group, the EB signal did not appear within the meningeal lymphatic vessels, although the structure of the meningeal lymphatic vessels seemed intact. This suggests a diminished functional CSF efflux through the meningeal lymphatic vessels in chronic hydrocephalus. As expected, peripheral lymphatic drainage to the deep cervical lymph nodes via the meningeal lymphatic vessels or other possible routes was also delayed in the CH group.

Building upon these findings, the broader implications of disrupted CSF dynamics in both experimental models and clinical conditions like CH (NPH) emphasize the profound interplay between CSF circulation and neurodegenerative processes. In NPH, altered CSF flow promotes the accumulation of neurotoxic metabolites, including amyloid beta (Aβ) and tau, which interfere with cellular function and accelerate neurodegenerative processes (Guzelcicek et al., 2021, Klemke et al., 2023). While transient motor improvements have been observed following enhanced CSF clearance in AD patients, unresolved dysfunction in CSF dynamics allows continued buildup of neurotoxic metabolites, sustaining cognitive decline (Klemke et al., 2023). Age-related declines in CSF flow and turnover further contribute to neurotoxin accumulation in NPH, compounding neurodegenerative progression (de Beer and Scheltens, 2016). Elevated neuroinflammatory markers and impaired CSF circulation are consistently observed in both NPH and neurodegenerative diseases (Braun et al., 2023). Ventricular enlargement observed in NPH patients demonstrates the harmful effects of hypoperfusion on CSF circulation, which disrupts waste clearance and accelerates neurodegenerative changes (Missori and Currà, 2015). Reduced cerebral perfusion and elevated intracranial pressure exacerbate oxidative stress, overwhelming antioxidant defenses and activating cellular stress responses that modify neurodegeneration-associated proteins (Guzelcicek et al., 2021). Dysfunction of the choroid plexus epithelium, which impairs Aβ clearance and promotes neuroinflammation, exacerbates neurodegenerative disease progression (Xie and Li, 2024). Collectively, these findings, together with our experimental results, reinforce the understanding of CSF dysfunction and its role in neurodegenerative diseases, offering valuable insights into their underlying mechanisms.

## 5 Conclusion

Our study evaluated several crucial steps in the CSF circulation pathway over time, including the glymphatic system, transependymal CSF flow, subarachnoid CSF flow, efflux through the venous sinus and meningeal lymphatic vessels, as well as peripheral lymph nodes drainage. Recognizing the role of the glymphatic system and its connection with cerebral lymphatic drainage, which functions as a clearance system for amyloid, tau, and other brain metabolic waste (Rasmussen et al., 2018, Reeves et al., 2020), clinical conditions such as chronic hydrocephalus or NPH may pose a significant risk for the progression of various neurodegenerative diseases, including Alzheimer’s disease. Therefore, it will be critical to conduct extensive research on the circulation of CSF and cerebral lymphatic drainage to identify risk factors and understand the pathophysiology of neurodegenerative diseases, which may lead to novel therapeutic strategies.

## Data Availability

The raw data supporting the conclusions of this article will be made available by the authors, without undue reservation.

## Footnotes

1
https://www.nc3rs.org.uk/arrive-guidelines

## References

[B1] Absinta M. Ha S. K. Nair G. Sati P. Luciano N. J. Palisoc M. (2017). Human and nonhuman primate meninges harbor lymphatic vessels that can be visualized noninvasively by MRI. *Elife* 6:e29738. 10.7554/eLife.29738 28971799 PMC5626482

[B2] Ahn J. H. Cho H. Kim J. H. Kim S. H. Ham J. S. Park I. (2019). Meningeal lymphatic vessels at the skull base drain cerebrospinal fluid. *Nature* 572 62–66. 31341278 10.1038/s41586-019-1419-5

[B3] Allbutt H. N. Henderson J. M. (2007). Use of the narrow beam test in the rat, 6-hydroxydopamine model of Parkinson’s disease. *J. Neurosci. Methods* 159 195–202. 16942799 10.1016/j.jneumeth.2006.07.006

[B4] Back D. B. Choi B. R. Han J. S. Kwon K. J. Choi D. H. Shin C. Y. (2020). Characterization of tauopathy in a rat model of post-stroke dementia combining acute infarct and chronic cerebral hypoperfusion. *Int. J. Mol. Sci.* 21:6929. 10.3390/ijms21186929 32967251 PMC7555397

[B5] Back D. B. Kwon K. J. Choi D. H. Shin C. Y. Lee J. Han S. H. (2017). Chronic cerebral hypoperfusion induces post-stroke dementia following acute ischemic stroke in rats. *J. Neuroinflammation* 14:216. 10.1186/s12974-017-0992-5 29121965 PMC5679180

[B6] Bloch O. Auguste K. I. Manley G. T. Verkman A. S. (2006). Accelerated progression of kaolin-induced hydrocephalus in aquaporin-4-deficient mice. *J. Cereb. Blood Flow Metab.* 26 1527–1537. 10.1038/sj.jcbfm.9600306 16552421

[B7] Bolte A. C. Shapiro D. A. Dutta A. B. Ma W. F. Bruch K. R. Kovacs M. A. (2023). The meningeal transcriptional response to traumatic brain injury and aging. *Elife* 12:e81154. 10.7554/eLife.81154 PMC9810333 36594818

[B8] Bradley W. G. (2015). CSF flow in the brain in the context of normal pressure hydrocephalus. *AJNR Am. J. Neuroradiol.* 36 831–838. 25355813 10.3174/ajnr.A4124 PMC7990574

[B9] Braun K. P. Van Eijsden P. Vandertop W. P. de Graaf R. A. Gooskens R. H. Tulleken K. A. (1999). Cerebral metabolism in experimental hydrocephalus: An in vivo 1H and 31P magnetic resonance spectroscopy study. *J. Neurosurg.* 91 660–668. 10.3171/jns.1999.91.4.0660 10507389

[B10] Braun M. Boström G. Ingelsson M. Kilander L. Löwenmark M. Nyholm D. (2023). Levels of inflammatory cytokines MCP-1, CCL4, and PD-L1 in CSF differentiate idiopathic normal pressure hydrocephalus from neurodegenerative diseases. *Fluids Barriers CNS* 20:72. 10.1186/s12987-023-00472-x 37833765 PMC10571396

[B11] Brinker T. Beck H. Klinge P. Kischnik B. Oi S. Samii M. (1998). Sinusoidal intrathecal infusion for assessment of CSF dynamics in kaolin-induced hydrocephalus. *Acta Neurochir.* 140 1069–1075. 10.1007/s007010050216 9856251

[B12] Chen L. J. Wang Y. J. Chen J. R. Tseng G. F. (2017). Hydrocephalus compacted cortex and hippocampus and altered their output neurons in association with spatial learning and memory deficits in rats. *Brain Pathol.* 27 419–436. 10.1111/bpa.12414 27411167 PMC8029119

[B13] Da Mesquita S. Louveau A. Vaccari A. Smirnov I. Cornelison R. C. Kingsmore K. M. (2018). Functional aspects of meningeal lymphatics in ageing and Alzheimer’s disease. *Nature* 560 185–191. 30046111 10.1038/s41586-018-0368-8 PMC6085146

[B14] de Beer M. H. Scheltens P. (2016). Cognitive decline in patients with chronic hydrocephalus and normal aging: ‘Growing into Deficits’. *Dement. Geriatr. Cogn. Dis Extra* 6 500–507. 10.1159/000450547 27920793 PMC5123026

[B15] Del Bigio M. R. Bruni J. E. (1988). Changes in periventricular vasculature of rabbit brain following induction of hydrocephalus and after shunting. *J. Neurosurg.* 69 115–120. 10.3171/jns.1988.69.1.0115 3223981

[B16] Edwards R. J. Dombrowski S. M. Luciano M. G. Pople I. K. (2004). Chronic hydrocephalus in adults. *Brain Pathol.* 14 325–336. 15446589 10.1111/j.1750-3639.2004.tb00072.x PMC8096062

[B17] Egawa T. Mishima K. Egashira N. Fukuzawa M. Abe K. Yae T. (2002). Impairment of spatial memory in kaolin-induced hydrocephalic rats is associated with changes in the hippocampal cholinergic and noradrenergic contents. *Behav. Brain Res.* 129 31–39. 10.1016/s0166-4328(01)00333-3 11809492

[B18] Gibbs W. N. Tanenbaum L. N. (2018). Imaging of hydrocephalus. *Appl. Radiol.* 47 5–13.

[B19] Guzelcicek A. Koyuncu I. Gönel A. Cigdem G. Karadag M. (2021). Relationship between oxidative stress, tau level and antioxidant mechanisms of the KEAP-1/NRF-2/HO-1 in children with hydrocephalus. *Antiinflamm Antiallergy Agents Med. Chem.* 20 282–289. 10.2174/1871523019666201228111713 33371862

[B20] Halperin J. J. Kurlan R. Schwalb J. M. Cusimano M. D. Gronseth G. Gloss D. (2015). Practice guideline: Idiopathic normal pressure hydrocephalus: Response to shunting and predictors of response: Report of the guideline development, dissemination, and implementation subcommittee of the american academy of neurology. *Neurology* 85 2063–2071. 26644048 10.1212/WNL.0000000000002193 PMC4676757

[B21] Hasan-Olive M. M. Enger R. Hansson H. A. Nagelhus E. A. Eide P. K. (2018). Loss of perivascular aquaporin-4 in idiopathic normal pressure hydrocephalus. *Glia* 67 91–100. 10.1002/glia.23528 30306658

[B22] Hwang Y. S. Shim I. Chang J. W. (2009). The behavioral change of locomotor activity in a kaolin-induced hydrocephalus rat model: Evaluation of the effect on the dopaminergic system with progressive ventricle dilatation. *Neurosci. Lett.* 462 198–202. 10.1016/j.neulet.2009.07.039 19616066

[B23] Jones H. Bucknall R. Harris N. (1991). The cerebral cortex in congenital hydrocephalus in the H-Tx rat: A quantitative light microscopy study. *Acta Neuropathol.* 82 217–224. 10.1007/BF00294448 1927278

[B24] Jung E. Gardner D. Choi D. Park E. Jin Seong Y. Yang S. (2017). Development and characterization of a novel Prox1-EGFP lymphatic and Schlemm’s canal reporter rat. *Sci. Rep.* 7:5577. 10.1038/s41598-017-06031-3 28717161 PMC5514086

[B25] Karimy J. K. Reeves B. C. Damisah E. Duy P. Q. Antwi P. David W. (2020). Inflammation in acquired hydrocephalus: Pathogenic mechanisms and therapeutic targets. *Nat. Rev. Neurol.* 16 285–296. 10.1038/s41582-020-0321-y 32152460 PMC7375440

[B26] Klemke L. L. Müller-Schmitz K. Kolman A. Seitz R. J. (2023). Evolution of neurodegeneration in patients with normal pressure hydrocephalus: A monocentric follow up study. *Neurol. Res. Pract.* 5:52. 10.1186/s42466-023-00272-6 37674250 PMC10483764

[B27] Klinge P. M. (2011). *Animals Models of Normal Pressure Hydrocephalus.* Berlin: Springer, 615–640.

[B28] Klinge P. M. Samii A. MuñHlendyck A. Visnyei K. Meyer G.-J. R. Walter G. F. (2003). Cerebral hypoperfusion and delayed hippocampal response after induction of adult kaolin hydrocephalus. *Stroke* 34 193–199. 10.1161/01.str.0000048820.17198.15 12511773

[B29] Kondziella D. LüDemann W. Brinker T. Sletvold O. Sonnewald U. (2002). Alterations in brain metabolism, CNS morphology and CSF dynamics in adult rats with kaolin-induced hydrocephalus. *Brain Res.* 927 35–41. 10.1016/s0006-8993(01)03320-0 11814430

[B30] Li J. Mcallister J. P. Shen Y. Wagshul M. E. Miller J. M. Egnor M. R. (2008). Communicating hydrocephalus in adult rats with kaolin obstruction of the basal cisterns or the cortical subarachnoid space. *Exp. Neurol.* 211 351–361. 18433747 10.1016/j.expneurol.2007.12.030

[B31] Louveau A. Filiano A. J. Kipnis J. (2018). Meningeal whole mount preparation and characterization of neural cells by flow cytometry. *Curr. Protoc. Immunol.* 121:e50. 10.1002/cpim.50 30008983 PMC6040815

[B32] Louveau A. Smirnov I. Keyes T. J. Eccles J. D. Rouhani S. J. Peske J. D. (2015). Structural and functional features of central nervous system lymphatic vessels. *Nature* 523 337–341. 26030524 10.1038/nature14432 PMC4506234

[B33] Magdoom K. N. Brown A. Rey J. Mareci T. H. King M. A. Sarntinoranont M. (2019). MRI of whole rat brain perivascular network reveals role for ventricles in brain waste clearance. *Sci. Rep.* 9:11480. 10.1038/s41598-019-44938-1 31391474 PMC6685961

[B34] McAllister J. P. Williams M. A. Walker M. L. Kestle J. R. Relkin N. R. Anderson A. M. (2015). An update on research priorities in hydrocephalus: Overview of the third national institutes of health-sponsored symposium “Opportunities for hydrocephalus research: Pathways to better outcomes”. *J. Neurosurg.* 123 1427–1438. 10.3171/2014.12.JNS132352 26090833

[B35] Missori P. Currà A. (2015). Progressive cognitive impairment evolving to dementia parallels parieto-occipital and temporal enlargement in idiopathic chronic hydrocephalus: A retrospective cohort study. *Front. Neurol.* 6:15. 10.3389/fneur.2015.00015 25759681 PMC4338750

[B36] Mu S. Ouyang L. Liu B. Zhu Y. Li K. Zhan M. (2011). Preferential interneuron survival in the transition zone of 3-NP-induced striatal injury in rats. *J. Neurosci. Res.* 89 744–754. 10.1002/jnr.22591 21337370

[B37] Nagra G. Li J. Mcallister J. Miller J. Wagshul M. Johnston M. (2008). Impaired lymphatic cerebrospinal fluid absorption in a rat model of kaolin-induced communicating hydrocephalus. *Am. J. Physiol. Regul. Integr. Comp. Physiol.* 294 R1752–R1759. 10.1152/ajpregu.00748.2007 18305019

[B38] Nagra G. Wagshul M. E. Rashid S. Li J. Mcallister J. P. Johnston M. (2010). Elevated CSF outflow resistance associated with impaired lymphatic CSF absorption in a rat model of kaolin-induced communicating hydrocephalus. *Cerebrospinal Fluid Res.* 7 1–8. 10.1186/1743-8454-7-4 20181144 PMC2831828

[B39] Nilsson O. R. Kari L. Rosenke R. Steele-Mortimer O. (2022). Protocol for RNA fluorescence in situ hybridization in mouse meningeal whole mounts. *STAR Protoc.* 3:101256. 10.1016/j.xpro.2022.101256 35345596 PMC8956821

[B40] Rasmussen M. K. Mestre H. Nedergaard M. (2018). The glymphatic pathway in neurological disorders. *Lancet Neurol.* 17 1016–1024. 30353860 10.1016/S1474-4422(18)30318-1 PMC6261373

[B41] Reeves B. C. Karimy J. K. Kundishora A. J. Mestre H. Cerci H. M. Matouk C. (2020). Glymphatic system impairment in Alzheimer’s disease and idiopathic normal pressure hydrocephalus. *Trends Mol. Med.* 26 285–295. 31959516 10.1016/j.molmed.2019.11.008 PMC7489754

[B42] Ringstad G. Vatnehol S. A. S. Eide P. K. (2017). Glymphatic MRI in idiopathic normal pressure hydrocephalus. *Brain* 140 2691–2705. 28969373 10.1093/brain/awx191 PMC5841149

[B43] Roussel-Queval A. Rebejac J. Eme-Scolan E. Paroutaud L. A. Rua R. (2023). Flow cytometry and immunohistochemistry of the mouse dural meninges for immunological and virological assessments. *STAR Protoc.* 4:102119. 10.1016/j.xpro.2023.102119 36853673 PMC9958090

[B44] Silverberg G. D. Mayo M. Saul T. Rubenstein E. Mcguire D. (2003). Alzheimer’s disease, normal-pressure hydrocephalus, and senescent changes in CSF circulatory physiology: A hypothesis. *Lancet Neurol.* 2 506–511. 12878439 10.1016/s1474-4422(03)00487-3

[B45] Silverberg G. D. Miller M. C. Machan J. T. Johanson C. E. Caralopoulos I. N. Pascale C. L. (2010). Amyloid and Tau accumulate in the brains of aged hydrocephalic rats. *Brain Res.* 1317 286–296. 10.1016/j.brainres.2009.12.065 20045398

[B46] Silverberg G. D. Miller M. C. Pascale C. L. Caralopoulos I. N. Agca Y. Agca C. (2015). Kaolin-induced chronic hydrocephalus accelerates amyloid deposition and vascular disease in transgenic rats expressing high levels of human APP. *Fluids Barriers CNS* 12:2. 10.1186/2045-8118-12-2 25685319 PMC4328504

[B47] Tarasoff-Conway J. M. Carare R. O. Osorio R. S. Glodzik L. Butler T. Fieremans E. (2015). Clearance systems in the brain-implications for Alzheimer disease. *Nat. Rev. Neurol.* 11 457–470. 10.1038/nrneurol.2015.119 26195256 PMC4694579

[B48] Ulfig N. Bohl J. Neudörfer F. Rezaie P. (2004). Brain macrophages and microglia in human fetal hydrocephalus. *Brain Dev.* 26 307–315. 10.1016/S0387-7604(03)00172-4 15165671

[B49] Wang Z. Zhang Y. Hu F. Ding J. Wang X. (2020). Pathogenesis and pathophysiology of idiopathic normal pressure hydrocephalus. *CNS Neurosci. Therapeutics* 26 1230–1240. 10.1111/cns.13526 PMC7702234 33242372

[B50] Xie S. Li F. (2024). Ependymal cells: Roles in central nervous system infections and therapeutic application. *J. Neuroinflammation* 21:255. 10.1186/s12974-024-03240-2 39385253 PMC11465851

[B51] Yankova G. Bogomyakova O. Tulupov A. (2021). The glymphatic system and meningeal lymphatics of the brain: New understanding of brain clearance. *Rev. Neurosci.* 32 693–705. 33618444 10.1515/revneuro-2020-0106

[B52] Yokota H. Vijayasarathi A. Cekic M. Hirata Y. Linetsky M. Ho M. (2019). Diagnostic performance of glymphatic system evaluation using diffusion tensor imaging in idiopathic normal pressure hydrocephalus and mimickers. *Curr. Gerontol. Geriatr. Res.* 2019:5675014. 10.1155/2019/5675014 31320896 PMC6609364

[B53] Zhang X. Chen X. P. Lin J. B. Xiong Y. Liao W. J. Wan Q. (2017). Effect of enriched environment on angiogenesis and neurological functions in rats with focal cerebral ischemia. *Brain Res.* 1655 176–185. 27818208 10.1016/j.brainres.2016.11.001

[B54] Zhou Y. Cai J. Zhang W. Gong X. Yan S. Zhang K. (2020). Impairment of the glymphatic pathway and putative meningeal lymphatic vessels in the aging human. *Ann. Neurol.* 87 357–369. 31916277 10.1002/ana.25670

